# Autoinducer2 affects trimethoprim‐sulfamethoxazole susceptibility in avian pathogenic *Escherichia coli* dependent on the folate synthesis‐associate pathway

**DOI:** 10.1002/mbo3.582

**Published:** 2018-02-09

**Authors:** Lumin Yu, Wenchang Li, Ming Zhang, Yunmei Cui, Xiaolin Chen, Jingtian Ni, Li Yu, Fei Shang, Ting Xue

**Affiliations:** ^1^ School of Life Sciences Anhui Agricultural University Hefei Anhui China; ^2^ School of Sciences Anhui Agricultural University Hefei Anhui China; ^3^ Department of Microbiology and Parasitology Anhui Key Laboratory of Zoonoses Anhui Medical University Hefei China

**Keywords:** autoinducer 2, avian pathogenic *Escherichia coli*, folate synthesis, trimethoprim‐sulfamethoxazole

## Abstract

Avian pathogenic *Escherichia coli* (APEC) causes airsacculitis, polyserositis, septicemia, and other mainly extraintestinal diseases in chickens, ducks, geese, pigeons, and other avian species, and is responsible for great economic losses in the avian industry. The autoinducer 2 (AI‐2) quorum sensing system is widely present in many species of gram‐negative and gram‐positive bacteria and has been proposed to be involved in interspecies communication. In clinical APEC strains, whether or not AI‐2 affects the expression of antibiotic‐related genes has not been reported. In this study, we have reported that exogenous AI‐2 increase the susceptibility of APEC strains to trimethoprim‐sulfamethoxazole (SXT) in a folate synthesis‐dependent pathway but not in the LsrR‐dependent manner. Our results further explained that exogenous AI‐2 can down regulate the transcription of the folate synthetase encoding genes *folA* and *folC*, and the folate synthesis‐related genes *luxS, metE,* and *metH*. Gel shift assays confirmed that LsrR, the AI‐2 receptor, did not bind to the promoters of *folA* and *folC*, suggesting that exogenous AI‐2 might influence folate metabolism by a feedback inhibition effect but not in the LsrR‐dependent pathway. This study might provide further information in the search for potential drug targets for prophylaxis of avian colibacillosis and for auxiliary antibiotics in the treatment of avian colibacillosis.

## 1 INTRODUCTION

Avian pathogenic *Escherichia coli* (APEC) is known to possess specific virulence attributes associated with colibacillosis in birds, and it is responsible for severe economic losses for the poultry industry worldwide (Dhomoulin & Fairbrother, 1999; Gross, 1994; Zhao et al., 2005). Avian colibacillosis can cause a variety of severe systemic and localized extraintestinal infections, with a complex syndrome characterized by multiple organ lesions like airsacculitis, pericarditis, perihepatitis, peritonitis, salpingitis, osteomyelitis, polyserositis, septicemia, and yolk sac infection (Dziva & Stevens, 2008; Ewers, Janssen, Kiessling, Philipp, & Wieler, 2004; Schouler et al., 2012; Wang et al., 2016). In the past few years, both the morbidity and mortality of APEC infections have increased rapidly and have become a major problem in the poultry industry (Altekruse et al., 2002; Blanco, Blanco, Mora, & Blanco, 1997). It has necessitated the use of antimicrobial chemotherapy to prevent and control outbreaks of APEC infections (Aggad, Ammar, Hammoudi, & Kihal, 2010; Watts, Salmon, Yancey, Nersessian, & Kounev, 1993).

A fixed‐dose combination of trimethoprim‐sulfamethoxazole (SXT) has been widely used as a broad‐spectrum antimicrobial agent since the 1960s (Church, Fitzgerald, Walker, Gibb, & Prendergast, 2015; Mcguinness, 1969). The rationale for the combination of trimethoprim with sulfamethoxazole is that each component blocks a different step in the folate biosynthetic pathway (Wormser, Keusch, & Heel, 1982). Sulfamethoxazole, a sulfonamide drug, is a structural analog of para‐aminobenzoic acid and competitively inhibits the synthesis of the intermediary dihydrofolic acid from its precursors, and trimethoprim is a structural analog of the pteridine portion of dihydrofolic acid that competitively inhibits dihydrofolate reductase and, consequently, decreases the production of the physiologically active tetrahydrofolic acid from dihydrofolic acid (Action, 2003; Church et al., 2015; Epstein, Amodio‐Groton, & Sadick, 1997). Although each agent alone is bacteriostatic, their blockade of two sequential enzymes results in bactericidal activity when combined (Action, 2003; Church et al., 2015). The synergy between trimethoprim and sulfamethoxazole together provides effective inhibition of enzymes involved in the bacterial synthesis of tetrahydrofolic acid, which is a necessary cofactor in bacterial nucleic acid synthesis (Grim, Rapp, Martin, & Evans, 2005; Sköld, 2011; Wormser et al., 1982). It has been reported that continuous intravenous SXT is a potential therapy to treat *E*. *coli* meningitis in rabbits and that the pharmacokinetic characteristics of sulfamethoxazole and trimethoprim given in combination to poultry produces a synergistic level for both antimicrobials and thus is considered to be a useful combination in the management of various avian diseases (Mylotte, Bates, Sergeant, Matson, & Jr, 1981; Queralt & Castells, 1985). The impact of SXT on *E*. *coli* in broiler intestinal or digestive tracts has already been reported (Dheilly et al., 2012). Furthermore, SXT has also been used in the treatment of bovine respiratory tract infections and feline and canine urinary tract infections caused by *E*. *coli* (Boothe, Smaha, Carpenter, Shaheen, & Hatchcock, 2012; Morrissey et al., 2016).

Quorum sensing (QS) is a process in which bacteria use chemical molecules as a signaling language to change behavior to adapt to specific environments (Bassler, 1999, 2002; Miller & Bassler, 2001). It has been reported that QS is a regulator of cellular processes such as bioluminescence production, virulence gene expression, cell division, biofilm formation, motility, metabolism, recombinant protein production, and the responsiveness to antibiotics (Ahmed, Petersen, & Scheie, 2007, 2009). Autoinducer 2 (AI‐2), a signal molecule, is produced to mediate both intra and interspecies communication and has the potential to influence both gene regulation and bacterial fitness. AI‐2 signals derive from spontaneous rearrangement of (4S)‐4, 5‐dihydroxy‐2, 3‐pentanedione (DPD) and are synthesized by a highly conserved AI synthase, LuxS, which is present in a variety of gram‐negative and gram‐positive bacteria species. In addition, AI‐2 supports cell population‐dependent behavior by interacting with central metabolism through the intracellular activated methyl cycle, which is related to the synthesis of tetrahydrofolic acid (Action, 2003; Bushby, 1973; Fuqua, Winans, & Greenberg, 1994; Vendeville, Winzer, Heurlier, Tang, & Hardie, 2005). It has been reported that AI‐2 increases the susceptibility in *Streptococcus anginosus* to ampicillin and erythromycin and decreases the susceptibility in *Staphylococcus aureus* to vancomycin, and AI‐2 is also involved in increased biofilm formation in *Streptococcus intermedius* (Ahmed et al., 2009; Xue, Zhao, & Sun, 2013). Our previous study confirmed that exogenous AI‐2 increased the antibiotic (such as ampicillin, oxacillin, and penicillin‐G) resistance of a clinical *E*. *coli* strain isolated from a dairy cow with mastitis by upregulating the expression of TEM‐type enzyme in an LsrR (the AI‐2 receptor)‐dependent manner (Xue et al., 2016). However, whether or not AI‐2 affects the expression of SXT susceptibility genes in APEC strains has not been reported.

In this study, we performed real‐time reverse transcription (RT)‐PCR experiments and digoxigenin (DIG) gel shift assays to evaluate how AI‐2 affects the SXT susceptibility of APEC. In this study, we reported that exogenous AI‐2 increses the susceptibility of APEC strains to trimethoprim‐sulfamethoxazole (SXT) in a folate synthesis‐dependent pathway but not in the LsrR‐dependent manner. Our results showed that exogenous AI‐2 can downregulate the transcription of the folate synthetase encoding genes *folA* and *folC*, and the folate synthesis‐related genes *luxS, metE,* and *metH*. Gel shift assays confirmed that LsrR, the AI‐2 receptor, did not bind to the promoters of *folA* and *folC*, suggesting that AI‐2 might influence folate metabolism by a feedback inhibition effect but not in the LsrR‐dependent pathway.

## 2 MATERIALS AND METHODS

### 2.1 Bacterial strains and culture conditions

The four *E*. *coli* strains used in this study were isolated from poultry, such as chickens, ducks, and pigeons (Table 1). These *E*. *coli* strains were stored at −80°C. Before each experiment, all strains were first cultured on Luria broth (LB) agar plates which contained 10 g/L Bacto tryptone (Oxoid, Basingstoke, UK), 5 g/L yeast extract (Oxoid, Basingstoke, UK), 10 g/L NaCl (Sangon, Shanghai, China), and 20 g/L agar power (Sangon, Shanghai, China) for 16 hr at 37°C in air supplied with 5% CO_2_. Colonies were then cultivated overnight in 2 ml of Mueller‐Hinton (MH) broth (Oxoid, Basingstoke, UK), named the first overnight cultures, and subsequently *E*. *coli* from the first overnight growth was diluted to an optical density at 600 nm (OD_600_) of approximately 0.03 in fresh MH broth for the following experiments. Cultures of all *E*. *coli* strains were grown at 37°C with shaking at 200 rpm. All the APEC strains used in this study were listed in Table 1.

**Table 1:** Strains used in this study and their susceptibility testing results

Strains	The host source	SXT MIC breakpoint (μg/ml)		MIC values (μg/ml)
		Susceptible	Resistant	
APEC 17	Chicken	≤2/38	≥4/76	0.5/9.5
APEC 19	Pigeon	≤2/38	≥4/76	2/38
APEC 29	Duck	≤2/38	≥4/76	2/38
APEC 40	Pigeon	≤2/38	≥4/76	2/38

### 2.2 Antimicrobial activity assay

The overnight cultures were inoculated into fresh MH broth and diluted to a final concentration of 0.03 by optical density at 600 nm, and 200 μl dilutions were dispensed into 96‐well plates (Costar, Corning, Steuben, NY, USA) containing SXT or the same volume of fresh MH medium with antibiotic‐free for control group, whereas the test group was with adding a final concentration of 39 μmol/L AI‐2. Every group was four technical replicates. The plates were incubated at 37°C for 16 hr, and then 10‐fold serial dilutions of cultures were performed by successive transfer (0.1 ml) through seven microfuge tubes containing 0.9 ml of MH. Next, 100 μl dilutions were dropped on Luria‐Bertani agar plates. After culturing for 16 hr at 37°C, viable colonies were counted and compared between the control and test groups via their colony‐forming units on LB agar plates. The survival rates of the control groups with or without exposure to SXT (without AI‐2) were designated as 100%. Experiments were repeated three times with four parallels.

### 2.3 Total RNA isolation, cDNA generation, and real‐time PCR processing

Overnight cultures of the four *E*. *coli* strains were diluted 1:100 in MH broth with or without SXT and, when necessary, AI‐2 was added for a final concentration of 39 μmol/L. The cultures were grown to the middle or late exponential phase at 37°C with shaking. Cells were collected and resuspended in RNase‐free water, and subsequently, total RNA was extracted from cells using the Trizol reagent (Ambion, Austin, TX), and residual DNA was removed using DNaseI (TaKaRa, Dalian, China). Real‐time RT‐PCR was performed using the PrimeScript 1st Strand cDNA synthesis kit, SYBR Premix Ex Taq (TaKaRa, Dalian, China), and a StepOne real‐time PCR system (Applied Biosystems, Carlsbad, CA). Differences in gene expression were calculated by ∆∆*C*_t_ (where *C*_t_ = cycle threshold) method, using the 16s *rDNA* gene as the housekeeping gene, normalized by subtracting the *C*_t_ value of 16s cDNA from target cDNA. All of the real‐time RT‐PCR assays were repeated at least three times with similar results. The primers used in this study are listed in Table 2, and the PCR amplification efficiency was controlled between 1.93 and 2.09.

**Table 2:** Oligonucleotide primers used in this study

Primer name	Oligonucleotide (5′‐3′)
g‐folA‐f	ATGATCAGTCTGATTGCGGCGTTAG
g‐folA‐r	TTACCGCCGCTCCAGAATCTCAAAG
g‐folC‐f	ATGATTATCAAACGCACTCCTCAAG
g‐folC‐r	TTACTTGCCACCGCTTCTCCTCGCG
g‐metE‐f	TGACAATATTGAATCACACCCTC
g‐metE‐r	ATTGTTCACCTTTCACTTTCCCC
g‐metH‐f	GTGAGCAGCAAAGTGGAACAACT
g‐metH‐r	TTTGGTGGACTCTCGATAAGCCG
rt‐16s‐f	TTTGAGTTCCCGGCC
rt‐16s‐r	CGGCCGCAAGGTTAA
rt‐lsrR‐f	CGGATCGCGTGGTTTT
rt‐lsrR‐r	TCAACATATGCGCCGC
rt‐folA‐f	GTAACGTGGGTGAAGTCGGT
rt‐folA‐r	TCAGCATCGTGGAATTCGCT
rt‐folC‐f	TGAAACCAGCGCCATTTGTG
rt‐folC‐r	CTGCTTGAACAGCCACAACG
rt‐luxS‐f	TCACAGTCGATCATACCCGG
rt‐luxS‐r	CTTCTTTGTTCGGCACGCAG
rt‐metE‐f	CCTGCGTCGCGAGCTGAAAA
rt‐metE‐r	GTTGATCCCAGTGACGAGCA
rt‐metH‐f	TGGAACAACTGCGTGCGCAG
rt‐metH‐r	AGTCGGCAAAGCGTTCACCA
p‐folA‐f	TAAAGAGTGACGTAAATC
p‐folA‐r	TGAGATTTCCCGATAAAA
p‐folC‐f	AATCTGCCAGCGCCGAAT
p‐folC‐r	GGTATCCGCTGATTCGTT

### 2.4 Gel shift assay

The transcriptional regulator LsrR protein was overexpressed and purified according to the methods described previously (Xue, Zhao, Sun, Zhou, & Sun, 2009). Two DNA fragments, p‐folA (191 bp) containing *folA* promoter and p‐folC (150 bp) containing *folC* promoter, were amplified by PCR from chromosomes of four APEC strains using two pairs of primers (p‐folA‐f, p‐folA‐r, p‐folC‐f, and p‐folC‐r) and pfu DNA polymerase (Promega, Madison, USA). The PCR products were purified by agarose gel electrophoresis. A digoxigenin (DIG) gel shift kit (Roche, Indianapolis, IN) was used for labeling of DNA fragments and detection of signals according to the manufacturer's instructions. Binding reactions were performed by incubating the labeled DNA fragments with various amounts of purified LsrR protein or the unlabeled DNA fragments at 25°C for 20 min in 4 μl of Binding buffer (5×). After incubation, 5 μl of loading buffer (5×) with bromophenol blue was added to the mixtures, which were then were electrophoresed in a 4% native polyacrylamide gel in 0.5× Tris‐borate EDTA buffer (45 mmol/L Tris‐borate and 1 mmol/L EDTA; pH 8.0). The band shifts were detected and analyzed according to the manufacturer's instructions.

### 2.5 Statistical analysis

The data were analyzed using the statistical software SPSS (ver. 19.0, IBM Corp., Armonk, NY) by a one‐way ANOVA method; the test results were shown as mean ± SD. The paired *t* test was used for statistical comparisons between groups. The level of statistical significance was set at a *p*‐value of ≤.05.

## 3 RESULTS

### 3.1 Exogenous AI‐2 increased the SXT susceptibility in APEC

To examine the susceptibility of 31 *E*. *coli* isolates (21, 3, 1, and 6 from chickens, ducks, geese, and pigeons with avian colibacillosis, respectively) to SXT, antimicrobial susceptibility testing was performed using the broth dilution technique. The minimal inhibitory concentrations (MIC) (Table 1) were determined and classified according to the guidelines of the Clinical Laboratory Standard Institute (CLSI). Strains APEC 17, APEC 19, APEC 29, and APEC 40 are representative of antibiotic susceptibility in all of the 31 APEC strains and were selected as the experimental subjects to perform the following test in this work. In addition, the SXT concentrations of strains APEC 17, APEC 19, APEC 29, and APEC 40 were selected (100/1900, 20/380, 20/380, and 2/38 ng/ml, respectively), as effective experimental concentrations inhibited the growth of the four APEC strains but according to their MIC values these were not considered to be lethal concentrations. The growth curves of four APEC strains were measured with the addition of exogenous AI‐2 in the absence or present of SXT to determine the effect of AI‐2 on cells growth of four APEC strains. These results illustrated that AI‐2 did not affect the growth of APEC in the absence of SXT (Figure S1), however, in the presence of SXT, addition of exogenous AI‐2 increased the susceptibility of APEC 17, APEC 19, APEC 29, and APEC 40 to SXT (Figure S2).

**Figure 1:**
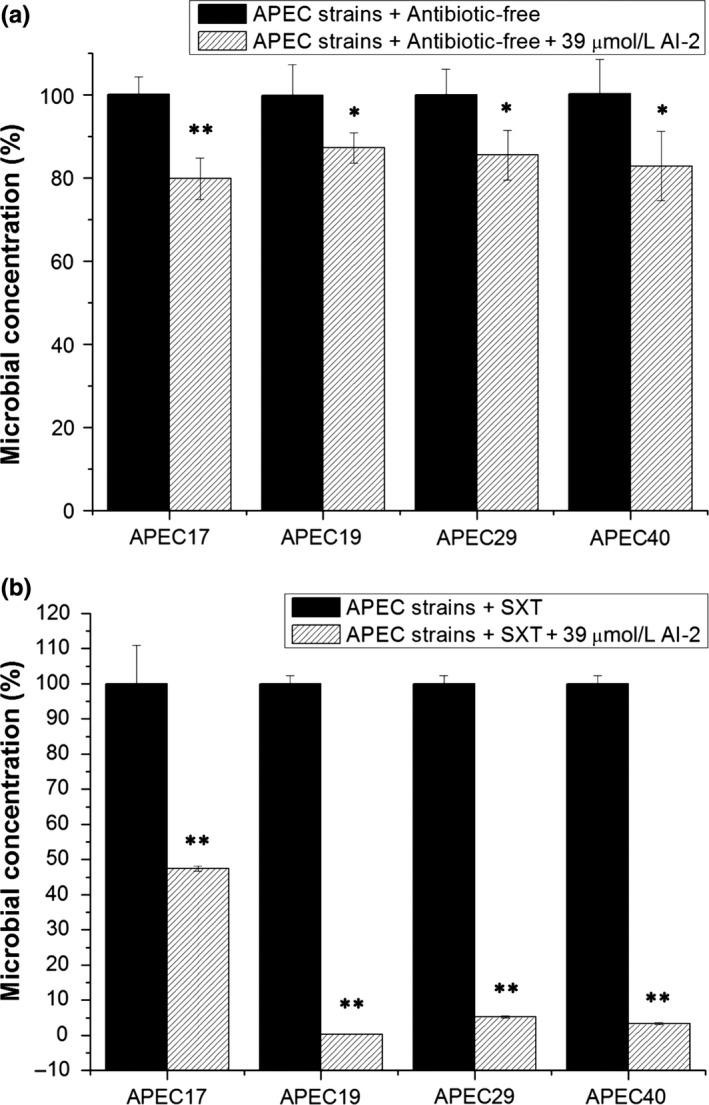
Colony‐forming units assays of the four APEC strains in the absence or presence of SXT. Colony‐forming units assays of APEC 17, APEC 19, APEC 29, APEC 40, and APEC 17, APEC 19, APEC 29, APEC 40 cultured with 39 μmol/L AI‐2 and with or without SXT: (a) without SXT, (b) with SXT: APEC 17 with 0.25/4.75 μg/ml, APEC 19 with 1/19 μg/ml, APEC 29 with 1/19 μg/ml, APEC 40 with 1/19 μg/ml. The survival rate of the control group without the addition of AI‐2 was designated 100%. The colony counts of the test group cultured with AI‐2 and SXT were compared to that of the control group (without AI‐2). Error bars indicate standard deviations. The results represent a mean of three independent experiments; **p <* *.01, *p <* *.05, indicating a difference between SXT alone and SXT + AI‐2

Antibacterial assays were performed to examine the changes in SXT susceptibility of APEC 17, APEC 19, APEC 29, and APEC 40 with the addition of exogenous AI‐2. This result showed that the survival rates of the test groups with AI‐2 were similar to that of the control groups (Figure 1a); however, decreased survival rates were observed in the test groups with exposure to SXT and AI‐2 compared to the control groups with SXT alone. The survival rates of APEC 17, APEC 19, APEC 29, and APEC 40 with SXT and AI‐2 were almost two times, 344 times, 19 times, and 30 times, respectively, lower than that of the control group with SXT alone (Figure 1b). These results indicated that with the addition of exogenous AI‐2, susceptibility of APEC 17, APEC 19, APEC 29, and APEC 40 to SXT was increased, suggesting that, AI‐2 might play an important role in the regulation of SXT susceptibility in these APEC isolates.

### 3.2 Exogenous AI‐2 increased SXT susceptibility by downregulating the folate synthetase encoding genes *folA* and *folC*

**Figure 2:**
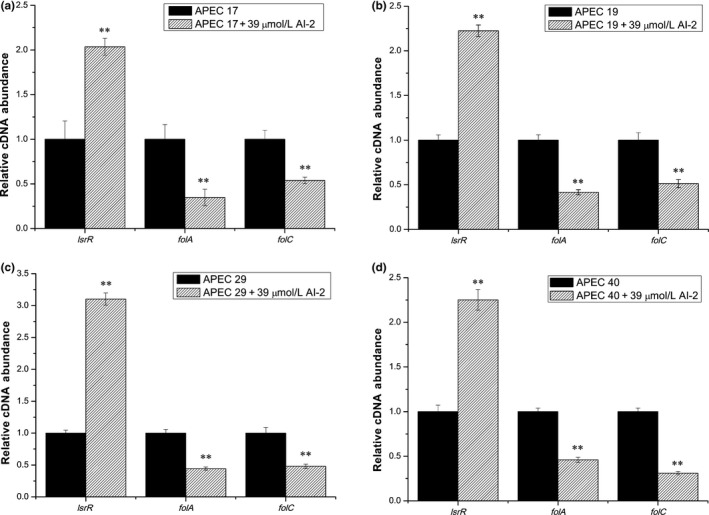
Comparative measurement of transcription of the folate synthetase encoding genes and the receptor encoding gene of AI‐2 in APEC strains without SXT. Comparative measurement of transcription (cDNA abundance) of *folA*,* folC,* and *lsrR* in APEC 17, APEC 19, APEC 29, and APEC 40. Relative transcript levels of *folA*,* folC,* and *lsrR* were tested by real‐time reverse transcription‐PCR in APEC 17, APEC 19, APEC 29, APEC 40, and APEC 17, APEC 19, APEC 29, APEC 40 cultured with 39 μmol/L AI‐2 in the absence of SXT: (a) APEC 17, (b) APEC 19, (c) APEC 29, and (d) APEC 40. Error bars indicate standard deviations. The results represent a mean of three independent experiments; **p <* *.01, indicating a difference between antibiotic‐free and AI‐2

**Figure 3:**
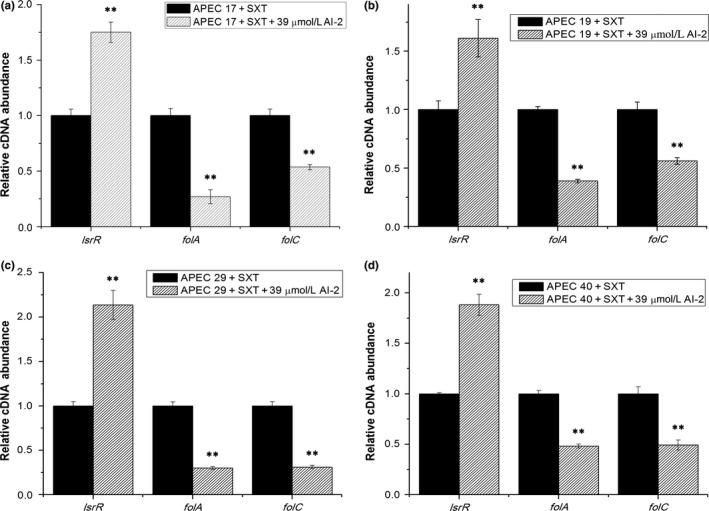
Comparative measurement of transcription of the folate synthetase encoding genes and the receptor encoding gene of AI‐2 in APEC strains with SXT. Comparative measurement of transcription (cDNA abundance) of *folA*,* folC,* and *lsrR* in APEC 17, APEC 19, APEC 29, and APEC 40. Relative transcript levels of *folA*,* folC,* and *lsrR* were tested by real‐time reverse transcription‐PCR in APEC 17, APEC 19, APEC 29, APEC 40, and APEC 17, APEC 19, APEC 29, APEC 40 cultured with 39 μmol/L AI‐2 in the presence of SXT: (a) APEC 17 with 50/950 ng/ml, (b) APEC 19 with 20/380 ng/ml, (c) APEC 29 with 20/380 ng/ml, and (d) APEC 40 with 20/380 ng/ml. Error bars indicate standard deviations. The results represent a mean of three independent experiments; **p <* *.01, indicating a difference between SXT and SXT+AI‐2

To investigate the mechanism of how AI‐2 affects SXT susceptibility in APEC, the transcript levels of genes associated with SXT susceptibility and AI‐2 QS were assessed by performing real‐time RT‐PCR experiments. It has already been reported that SXT could lead to bacterial death by affecting the folate metabolic pathway and *folA* and *folC* are two important encoding genes in folate metabolism. The transcript levels of *folA, folC,* and *lsrR*, which encode dihydrofolate reductase (DHFR), dihydrofolate synthetase (DHFS), and the receptor of AI‐2, respectively, were measured in the four APEC strains under different conditions. As shown in Figure 2, the transcript levels of *lsrR* were increased 2.0‐fold, 2.2‐fold, 3.1‐fold, and 2.2‐fold by the addition of exogenous AI‐2 in the absence of SXT in APEC 17, APEC 19, APEC 29, and APEC 40, respectively. Accordingly, the transcript levels of *folA* were decreased 2.9‐fold, 2.4‐fold, 2.3‐fold, and 2.2‐fold, and the transcript levels of *folC* were decreased 1.9‐fold, 1.9‐fold, 2.1‐fold, and 3.2‐fold in APEC 17, APEC 19, APEC 29, and APEC 40, respectively. Furthermore, in the presence of SXT, as shown in Figure 3, the addition of AI‐2 resulted in 1.8‐fold, 1.6‐fold, 2.1‐fold, and 1.9‐fold increases in the transcription of *lsrR*, 3.7‐fold, 2.6‐fold, 3.3‐fold, and 2.1‐fold decreases in the transcription of *folA*, and 1.9‐fold, 1.8‐fold, 3.2‐fold, and 2.0‐fold decreases in the transcription of *folC* in APEC 17, APEC 19, APEC 29, and APEC 40, respectively. These results suggested that exogenous AI‐2 might downregulate the transcription of *folA* and *folC* and influence folate metabolism in an LsrR‐dependent pathway.

### 3.3 LsrR did not bind to the promoters of *folA* and *folC*

**Figure 4:**
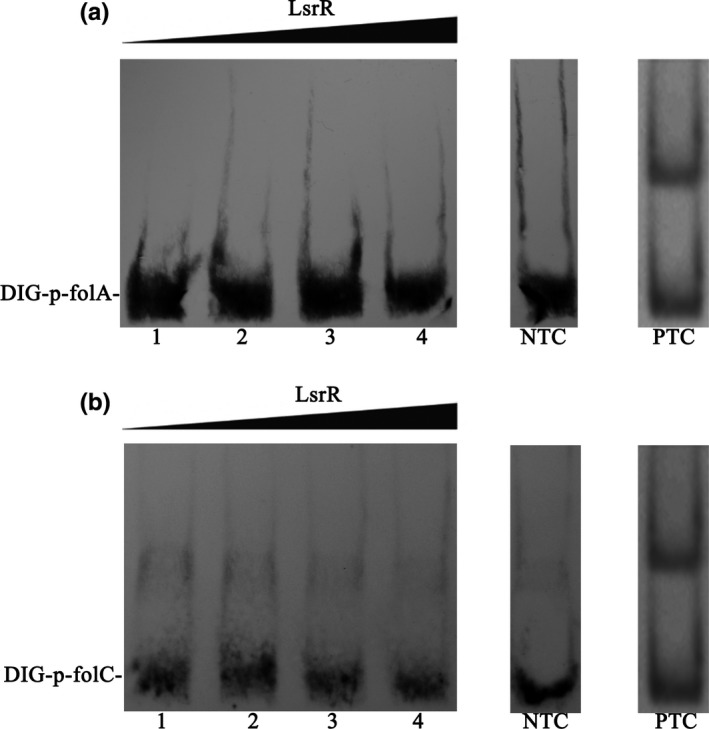
The binding ability of LsrR to the *folA* and *folC* promoters were determined by gel‐shift assays. Increasing amounts of LsrR were incubated with DIG‐labeled *folA* and *folC* promoters (DIG‐p‐folA and DIG‐p‐folC). In each panel, from lane 1 to 5, the amounts of LsrR were 0, 0.5, 1, 2, and 2 pmol, respectively; 200 fmol of DIG‐labeled probes was added to all lanes. NTC, negative control, except for the labeled probes, 1 pmol of unlabeled probes was incubated with the LsrR protein as the competitive control. PTC, positive control, the binding ability of LsrR to the *TEM* promoter. (a) the *folA* promoter and (b) the *folC* promoter

It has been reported that LsrR contains a helix‐turn‐helix (HTH) DNA binding domain and is a QS regulator that can regulate the transcription of a variety of genes by binding to their promoters. On the basis of these findings, we hypothesized that exogenous AI‐2 might activate LsrR, which then inhibits the transcription of *folA* and *folC* by directly binding to their promoter regions. To determine whether or not LsrR can bind to the promoters of *folA* and *folC*, we performed DIG‐gel shift assays. The results showed that LsrR can not bind to the promoters of *folA* and *folC* (Figure 4), indicating that AI‐2 might influence folate metabolism in an LsrR‐independent pathway.

### 3.4 Exogenous AI‐2 influenced folate metabolism by downregulating the transcription of the folate synthesis‐related genes *luxS*,* metE,* and *metH* in APEC

**Figure 5:**
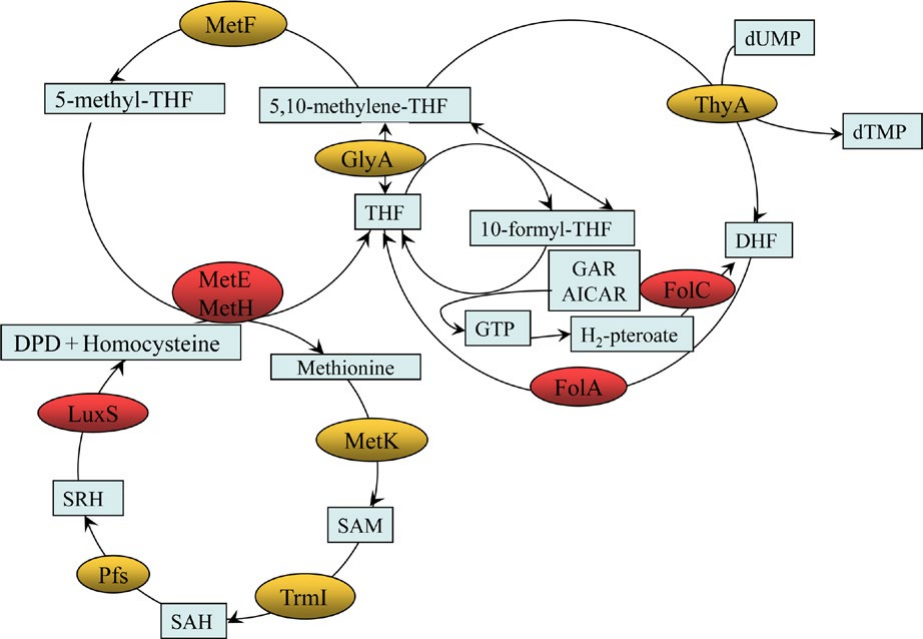
The folate synthesis‐associate pathways. DHF, dihydrofolate; FolC, dihydrofolate synthase; FolA, dihydrofolate reductase; THF, tetrahydrofolate; SAH, S‐adenosylhomocysteine; SAM, S‐adenosylmethionine; SRH, S‐ribosylhomocysteine; GlyA, serine hydroxymethyltransferase; MetF, 5,10‐methylenetetrahydrofolate reductase; AICAR, 5‐aminoimidazole‐4‐carboxamine ribonucleotide; dTMP, deoxythymidine monophosphate; dUMP, deoxyuridine monophosphate; GAR, glycinamide ribonucleotide; GTP, guanosine triphosphate; ThyA, thymidylate synthase; MetE, methionine synthase; MetH, B12‐dependent homocysteine‐5‐methyltetrahydrofolate methyltransferase; MetK, S‐ adenosylmethionine synthase; TrmI, SAM‐dependent methylase; Pfs, S‐adenosylhomocysteine nucleosidase; LuxS, S‐ribosylhomocysteinase; DPD, 4,5‐dihydroxy‐2,3‐pentanedione, the precursor of AI‐2

**Figure 6:**
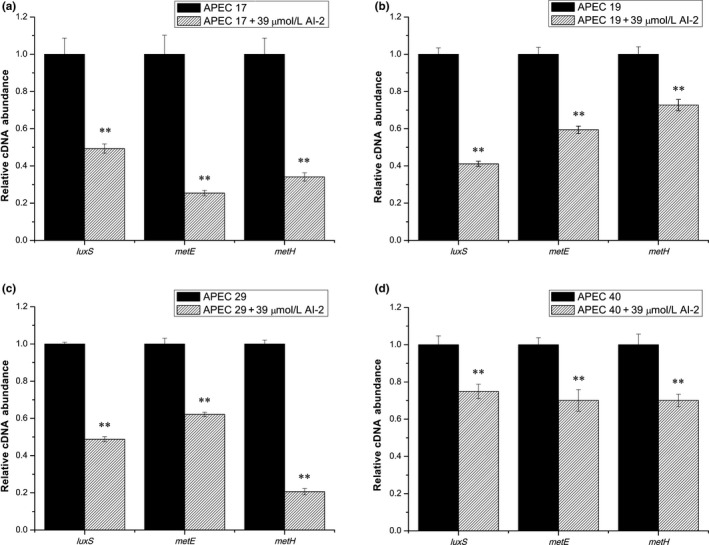
Comparative measurement of transcription of the folate synthesis‐related genes in APEC strains without SXT. Comparative measurement of transcription (cDNA abundance) of *luxS*,* metE,* and *metH* in APEC 17, APEC 19, APEC 29, and APEC 40. Relative transcript levels of *luxS*,* metE,* and *metH* were tested by real‐time reverse transcription‐PCR in APEC 17, APEC 19, APEC 29, APEC 40, and APEC 17, APEC 19, APEC 29, APEC 40 cultured with 39 μmol/L AI‐2 in the absence of SXT: (a) APEC 17, (b) APEC 19, (c) APEC 29, and (d) APEC 40. Error bars indicate standard deviations. The results represent a mean of three independent experiments; **p <* *.01, indicating a difference between antibiotic‐free and AI‐2

**Figure 7:**
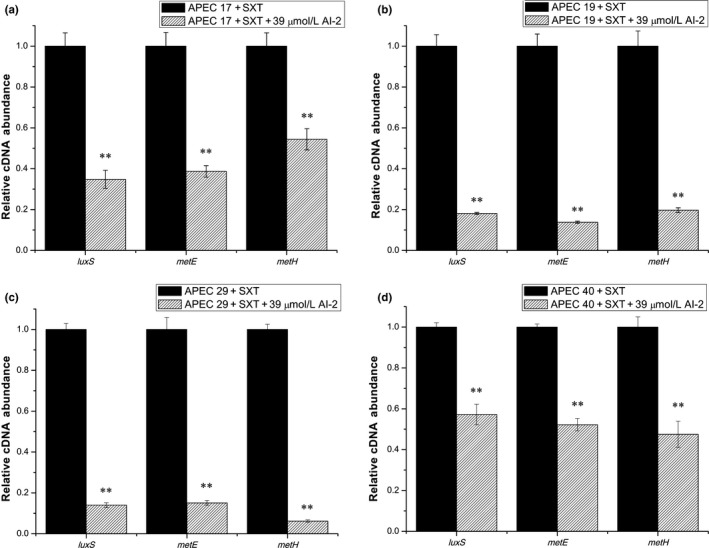
Comparative measurement of transcription of the folate synthesis‐related genes in APEC strains with SXT. Comparative measurement of transcription (cDNA abundance) of *luxS*,* metE,* and *metH* in APEC 17, APEC 19, APEC 29, and APEC 40. Relative transcript levels of *luxS*,* metE,* and *metH* were tested by real‐time reverse transcription‐PCR in APEC 17, APEC 19, APEC 29, APEC 40, and APEC 17, APEC 19, APEC 29, APEC 40 cultured with 39 μmol/L AI‐2 in the presence of SXT: (a) APEC 17 with 50/950 ng/ml, (b) APEC 19 with 20/380 ng/ml, (c) APEC 29 with 20/380 ng/ml, and (d) APEC 40 with 20/380 ng/ml. Error bars indicate standard deviations. The results represent a mean of three independent experiments; **p <* *.01, indicating a difference between SXT and SXT + AI‐2

It has been reported that *luxS* (encodes S‐ribosylhomocysteinase), *metE* (encodes B_12_‐dependent homocysteine‐5‐methyltetrahydrofolate methyltransferase, MTR), and *metH* (encodes methionine synthase, MS) are related to the folate synthesis pathway, and AI‐2 is one of the products of LuxS (Figure 5). We speculated that exogenous AI‐2 might inhibit the transcription of *luxS* by a feedback inhibition effect, and then decrease the amount of homocysteine in the APEC strains, and finally, influence the transcription of other relative genes in folate metabolism. To prove this hypothesis, the transcript levels of *luxS*,* metH,* and *metE* were measured in the four APEC strains under different conditions. As shown in Figure 6, the transcript levels of *luxS* were decreased 2.0‐fold, 2.4‐fold, 2.0‐fold, and 1.3‐fold, the transcript levels of *metE* were decreased 3.9‐fold, 1.7‐fold, 1.6‐fold, and 1.4‐fold, and the transcript levels of *metH* were decreased 2.9‐fold, 1.4‐fold, 4.9‐fold, and 1.4‐fold by the addition of exogenous AI‐2 in the absence of SXT in APEC 17, APEC 19, APEC 29, and APEC 40, respectively. Furthermore, in the presence of SXT, as shown in Figure 7, the addition of AI‐2 resulted in 2.9‐fold, 5.5‐fold, 7.1‐fold, and 1.7‐fold decreases in transcription of *luxS*, 2.6‐fold, 7.2‐fold, 6.6‐fold, and 1.9‐fold decreases in transcription of *metE,* and 1.8‐fold, 5.1‐fold, 16.1‐fold, and 2.1‐fold decreases in transcription of *metH* in APEC 17, APEC 19, APEC 29, and APEC 40, respectively. These results confirmed that exogenous AI‐2 can affect folate synthesis by downregulating the transcription of *luxS*,* metE*,* metH, folA, and folC,* which then affects the SXT susceptibility in APEC strains by a folate synthesis‐associated pathway.

## 4 DISCUSSION

In recent years, the QS system has emerged as a topic of great interest due to its involvement in various biochemical processes. Previous studies reported that AI‐2 is an important QS signal molecule in gram‐negative and ‐positive bacteria, including *E*. *coli*, suggesting that AI‐2 could play an important role in the physiological activity or regulation of *E*. *coli* (Ahmed et al., 2009; Vendeville et al., 2005; Xue et al., 2009). In *E*. *coli* model strains, the functional mechanisms of AI‐2 have been well studied (Wang, Li, March, Valdes, & Bentley, 2005; Xue et al., 2009). However, these studies did not involve the antibiotic resistance‐ or susceptibility‐associated genes. Our previous study demonstrated that exogenous AI‐2 increased the antibiotic (such as ampicillin, oxacillin, and penicillin‐G) resistance of a clinical *E*. *coli* strain isolated from a dairy cow with mastitis by upregulating the expression of resistance gene *TEM*, and the regulation was directly related to LsrR which is the receptor of AI‐2 (Xue et al., 2016). However, in avian pathogenic *E*. *coli*, whether or not AI‐2 affects antibiotic‐related genes and pathogenic genes has not been reported. In this study, we found that, in the presence of SXT, exogenous AI‐2 decreased the physiological activity of APEC, and increased the susceptibility of APEC to SXT by an LsrR‐independent pathway. Therefore, we have confirmed that exogenous AI‐2 affects SXT susceptibility in APEC strains by a folate synthesis‐related pathway, indicating that the combined use of AI‐2 and SXT can minimize the dose of SXT in the treatment.

Previous studies on AI‐2 did not explain its metabolic effect on APEC. This study indicated that AI‐2 increases the SXT susceptibility of APEC in a folate‐dependent manner. The addition of exogenous pre‐AI‐2 molecule DPD triggers a product feedback inhibition effect, which then decreases the expression of *luxS* and the amount of the other products of LuxS, such as homocysteine (Figure 5). Homocysteine is the substrate of MetE and MetH, which are important enzymes in the folate synthesis pathway. The substrate inhibition effect caused by the decrease in homocysteine downregulates the expression of *metE* and *metH*, and then results in a decrease in the intermediate metabolite tetrahydrofolate (THF), which is an important substrate used to synthesize purine and pyrimidine. In THF metabolism, purine and pyrimidine are two important intermediate metabolites used to synthesize THF again, and *folA* and *folC* are two important folate synthetase encoding genes. In the absence of SXT, AI‐2 down‐regulated the transcript levels of folate synthetase encoding genes *folA* and *folC* through the folate cycle pathway only, and did not affect the survival rates of the test groups (with AI‐2) compared to the control groups. However, in the presence of SXT, exogenous AI‐2 strengthened the growth inhibition of SXT on the APEC strains by downregulating the transcript levels of folate‐associated genes. This study might provide further information in the search for potential drug targets for prophylaxis of avian colibacillosis and for auxiliary antibiotics in the treatment of avian colibacillosis.

## CONFLICT OF INTEREST

The authors report no conflicts of interest in this work.
