# Supplementary figures and images for: Autoinducer2 affects trimethoprim‐sulfamethoxazole susceptibility in avian pathogenic Escherichia coli dependent on the folate synthesis‐associate pathway
Source: Microbiologyopen. 2018 Feb 9;7(4):e00582. doi: 10.1002/mbo3.582 (PMC6079169; doi:10.1002/mbo3.582)

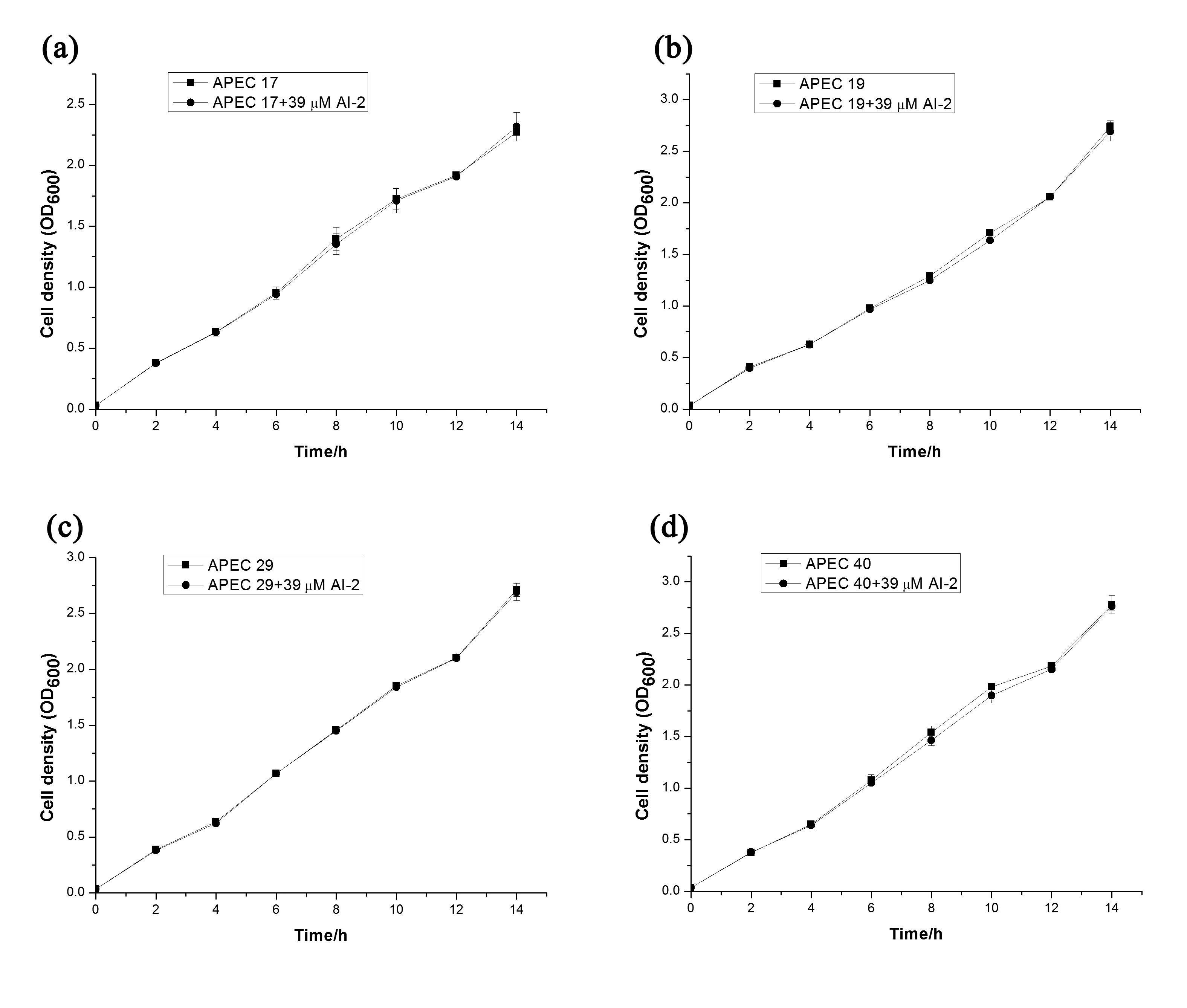

Supplement: Supplementary file 1 [file MBO3-7-e00582-s001.tif]

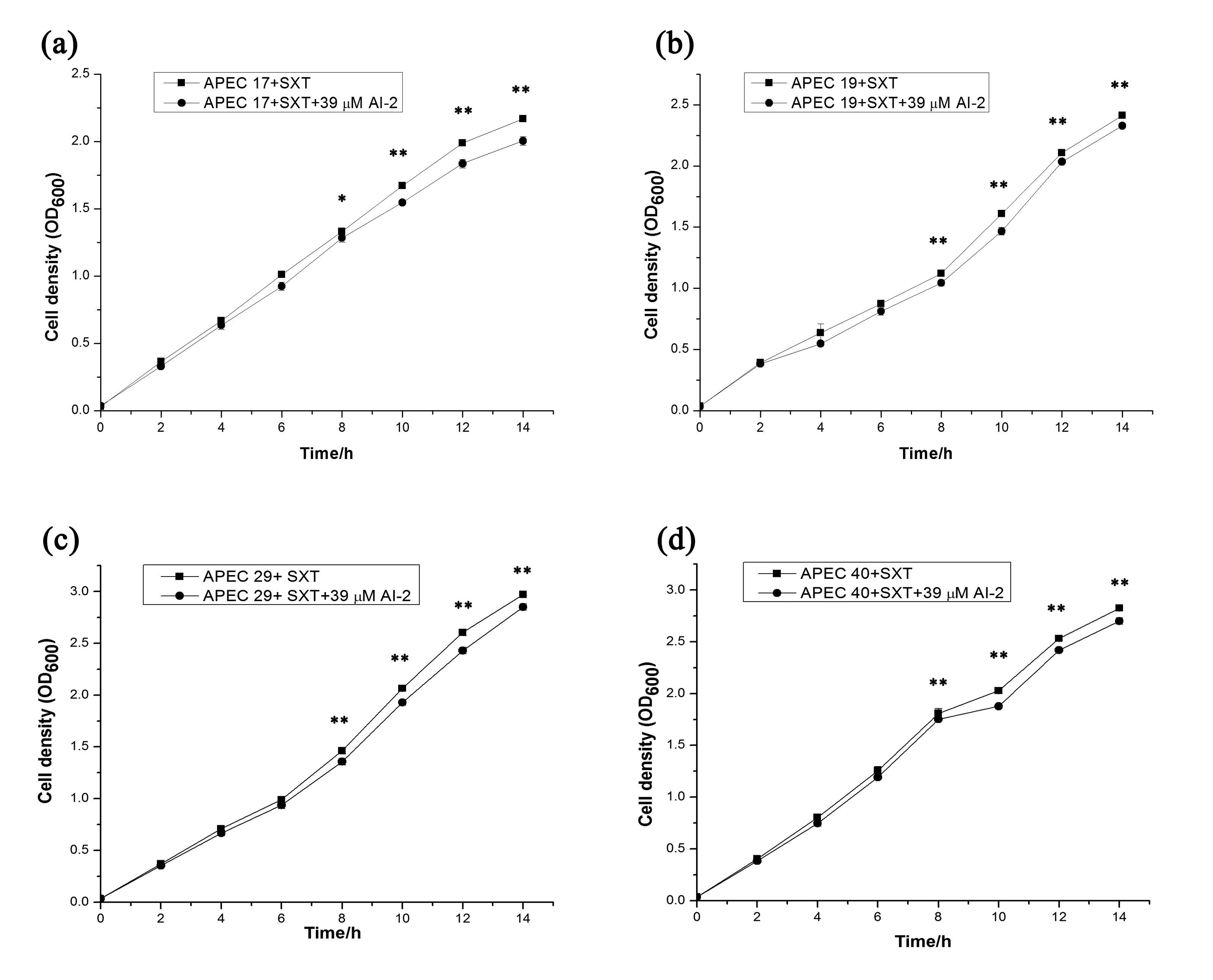

Supplement: Supplementary file 2 [file MBO3-7-e00582-s002.tif]
