# Supplementary material for: Autoinducer2 affects trimethoprim‐sulfamethoxazole susceptibility in avian pathogenic Escherichia coli dependent on the folate synthesis‐associate pathway
Source: Microbiologyopen. 2018 Feb 9;7(4):e00582. doi: 10.1002/mbo3.582 (PMC6079169; doi:10.1002/mbo3.582)
Supplement: Supplementary file 3 [file MBO3-7-e00582-s003.doc]

**Supplemental Material**

**Bacterial Growth Assay**

The overnight cultures of the four *E. coli* strains were diluted to a final concentration of approximately 7×10^6^ cfu/mL, or OD_600_ of approximately 0.03, in MH broth with different final concentrations of SXT, and were incubated at 37 ℃ with shaking. When necessary, the pre-AI-2 molecule DPD (Omm Scientific Inc., TX, USA) was added for a final concentration of 39 µM. The cell density was detected at each time point by using a DU730 spectrophotometer (Beckman Coulter, Miami, FL, USA).
