# Supplementary material for: Autoinducer2 affects trimethoprim‐sulfamethoxazole susceptibility in avian pathogenic Escherichia coli dependent on the folate synthesis‐associate pathway
Source: Microbiologyopen. 2018 Feb 9;7(4):e00582. doi: 10.1002/mbo3.582 (PMC6079169; doi:10.1002/mbo3.582)
Supplement: Supplementary file 4 [file MBO3-7-e00582-s004.docx]

**SUPPLEMENTARY FIGURE LEGENDS**

**FIGURE S1** The growth curves of the four APEC strains with AI-2 in the absence of SXT. The growth curves (measured as optical density at 600 nm, OD_600_) of APEC 17, APEC 19, APEC 29, APEC 40 and APEC 17, APEC 19, APEC 29, APEC 40 cultured with 39 μM AI-2 in the absence of SXT: **(a)** APEC 17, **(b)** APEC 19, **(c)** APEC 29, and **(d)** APEC 40. Error bars indicate standard deviations. The results represent a mean of three independent experiments.

**FIGURE S2** The growth curves of the four APEC strains with AI-2 in the presence of SXT. The growth curves (measured as optical density at 600 nm, OD_600_) of APEC 17, APEC 19, APEC 29, APEC 40 and APEC 17, APEC 19, APEC 29, APEC 40 cultured with 39 μM AI-2 in the presence of SXT: **(a)** APEC 17 with 100/1900 ng/mL SXT, **(b)** APEC 19 with 20/380 ng/mL SXT, **(c)** APEC 19 with 20/380 ng/mL SXT, and **(d)** APEC 40 with 2/38 ng/mL SXT. Error bars indicate standard deviations. The results represent a mean of three independent experiments; **P < 0.01, *P < 0.05, indicating a difference between SXT alone and SXT + AI-2.
